# Integrated Pangenome Analysis and Pharmacophore Modeling Revealed Potential Novel Inhibitors against *Enterobacter xiangfangensis*

**DOI:** 10.3390/ijerph192214812

**Published:** 2022-11-10

**Authors:** Mohammed S. Almuhayawi, Soad K. Al Jaouni, Samy Selim, Dalal Hussien M. Alkhalifah, Romina Alina Marc, Sidra Aslam, Peter Poczai

**Affiliations:** 1 Department of Medical Microbiology and Parasitology, Faculty of Medicine, King Abdulaziz University, Jeddah 21589, Saudi Arabia; 2 Department of Hematology/Oncology, Yousef Abdulatif Jameel Scientific Chair of Prophetic Medicine Application, Faculty of Medicine, King Abdulaziz University, Jeddah 21589, Saudi Arabia; 3 Department of Clinical Laboratory Sciences, College of Applied Medical Sciences, Jouf University, Sakaka 72388, Saudi Arabia; 4 Department of Biology, College of Science, Princess Nourah bint Abdulrahman University, P.O. Box 84428, Riyadh 11671, Saudi Arabia; 5 Food Engineering Department, Faculty of Food Science and Technology, University of Agricultural Science and Veterinary Medicine Cluj-Napoca, 3-5 Calea Mănă ¸stur Street, 400372 Cluj-Napoca, Romania; 6 Department of Bioinformatics and Biotechnology, Government College University Faisalabad, Punjab 38000, Pakistan; 7 Banner Sun Health Research Institute, Sun City, AZ 85351, USA; 8 Botany Unit, Finnish Museum of Natural History, University of Helsinki, P.O. Box 7, FI-00014 Helsinki, Finland

**Keywords:** *Enterobacter xiangfangensis*, antibiotic-resistant, infection, in silico, therapeutic target, virulent, subtractive proteomic

## Abstract

*Enterobacter xiangfangensis* is a novel, multidrug-resistant pathogen belonging to the Enterobacter genus and has the ability to acquire resistance to multiple antibiotic classes. However, there is currently no registered *E. xiangfangensis* drug on the market that has been shown to be effective. Hence, there is an urgent need to identify novel therapeutic targets and effective treatments for *E. xiangfangensis*. In the current study, a bacterial pan genome analysis and subtractive proteomics approach was employed to the core proteomes of six strains of *E. xiangfangensis* using several bioinformatic tools, software, and servers. However, 2611 nonredundant proteins were predicted from the 21,720 core proteins of core proteome. Out of 2611 nonredundant proteins, 372 were obtained from Geptop2.0 as essential proteins. After the subtractive proteomics and subcellular localization analysis, only 133 proteins were found in cytoplasm. All cytoplasmic proteins were examined using BLASTp against the virulence factor database, which classifies 20 therapeutic targets as virulent. Out of these 20, 3 cytoplasmic proteins: ferric iron uptake transcriptional regulator (FUR), UDP-2,3diacylglucosamine diphosphatase (UDP), and lipid-A-disaccharide synthase (lpxB) were chosen as potential drug targets. These drug targets are important for bacterial survival, virulence, and growth and could be used as therapeutic targets. More than 2500 plant chemicals were used to molecularly dock these proteins. Furthermore, the lowest-binding energetic docked compounds were found. The top five hit compounds, *Adenine*, *Mollugin*, *Xanthohumol C*, *Sakuranetin,* and *Toosendanin* demonstrated optimum binding against all three target proteins. Furthermore, molecular dynamics simulations and MM/GBSA analyses validated the stability of ligand–protein complexes and revealed that these compounds could serve as potential *E. xiangfangensis* replication inhibitors. Consequently, this study marks a significant step forward in the creation of new and powerful drugs against *E. xiangfangensis*. Future studies should validate these targets experimentally to prove their function in *E. xiangfangensis* survival and virulence.

## 1. Introduction

Enterobacter is a genus of facultatively anaerobic, rod-shaped, Gram-negative bacteria of the Enterobacteriaceae family that is mainly associated with healthcare-related infections. Currently, there are 22 different types of Enterobacter [1]. Many previously successful antibiotics have become increasingly ineffective against Enterobacter. The primary mechanism of antibiotic resistance in Enterobacter species is the presence of beta-lactamases. Beta-lactamases can hydrolyze the beta-lactam ring found in cephalosporins and penicillin. The existence of this enzyme has contributed to an increase in resistant Enterobacter pathogens [2]. The World Health Organization published a list of bacteria that were resistant to medicines in 2017, and carbapenem-resistant enterobacteriaceae was included in the critical priority group for the urgent need to discover new antibiotics [3].

*Enterobacter xiangfangensis* is a motile, Gram-negative bacterium with a size of 0.8–1 1–1.5 m. It is a common pathogen in China [4]. Many hospital-acquired infections are caused by *E. xiangfangensis*, which has a high level of resistance to broad-spectrum antibiotics [5]. The bacteria can also obtain carbapenemase genes from other Enterobacter species, according to reports [6]. There is no appropriate vaccination for *E. xiangfangensis*, which exacerbates the global issue [7]. Recommendations, such as adapting antibiotic management programs and improving diagnostic decision-making processes, and follow-up can increase the efficacy of infectious disease therapy and slow the emergence of bacterial resistance [8]. Hence, the development of an effective treatment strategy for *E. xiangfangensis*, as well as potent drugs and novel therapeutic targets, is critical.

Traditional methods for finding new drugs are costly and time-consuming, but newer technology has overcome these drawbacks. Through the use of computational analytic techniques, such as core genome and subtractive genomics, the modern genomic era has made it possible to search for potential therapeutic targets at the genome level in bacteria [9]. Subtractive genomic and core genome approaches have been developed to discover the core essential genomes that are distinct from the human genome [10,11,12] and further integration with bioinformatics provided much better results [11,13,14]. These methods have been used to combat a variety of human pathogens, including *Shigella sonnei*, *Staphylococcus aureus*, *Mycoplasma pneumonia*, *Streptococcus Pyogenes*, *Staphylococcus saprophyticus*, and *Chlamydia trachomatis* [15,16,17,18,19,20]. This research will use in silico methods to connect the proteome and genomic data of the *E. xiangfangensis* species in order to pinpoint potential drugs. It can be used to classify effective inhibitors, assisting in the discovery of drugs that can limit pathogenic progression [21]. A pan genome approach was used in the current study to compare the proteomes from the six *E. xiangfangensis* genomes, and only the genes that were shared by all *E. xiangfangensis* strains were chosen. The core genome was subsequently filtered based on bacterial essentiality and host nonhomology. Among these proteins, cytoplasmic proteins were found to be good drug targets. A library of 2500 plant compounds was used for virtual screening on these nonhost homologous and essential protein targets. The proposed innovative lead druggable compounds that can bind to the indicated target proteins can then be produced based on the identified putative targets.

## 2. Materials and Methods

### 2.1. Retrieval and Pan Genome Analysis of Bacterial Proteome

*E. xiangfangensis* proteomes were downloaded from the NCBI database and then subjected to OrthoFinder program (University of Oxford, Oxford, UK) without altering the default parameters [22]. OrthoFinder performs calculations based on BLAST searches. So, internal scripts were developed for the finding of core genes in all understudied strains. The core sequences were then taken into consideration for additional downward analysis. Previously isolated *E. xiangfangensis* (isolated from ear, blood, urine, and sputum) was kept in frozen (−9 °C) stocks that had been provided with 20% (*v*/*v*) glycerol. The strains were identified using different tests, such as the Gram reaction, cell morphology, and catalase assays. 16S rDNA sequencing analysis and the API 50 CHL test (bio-Merieux, Marcy-l’Étoile, France) were used to identify the strains

### 2.2. Redundancy Analysis and Identification of Essential Proteins

Paralogous genes, which are duplicated genes, are typically not necessary for the development of drug. The CD-HIT web server was employed at 80% efficiency to eliminate redundant proteins and extract non-redundant core sequences [23]. It is believed that essential proteins are the basis of life and are required by organisms for their survival. Essential proteins were obtained through the use of the Geptop 2.0 server [24].

### 2.3. Homology Analysis and Subcellular Localization

The thresholds used were bits score of less than 100 and an e-value less than 0.0001, and the BLOSUM 62 matrix was chosen. Using BLASTp, a tool in NCBI-BLAST that identifies nonhomologous sequences, the proteome of Homo sapiens and the essential proteins of *E. xiangfangensis* were compared [25]. Predicting a protein’s precise subcellular location is a straightforward and very inexpensive method to learn about its function. Furthermore, because proteins can be located at various places, localization is a key part of creating any therapeutic agent. Subcellular localization of proteins from *E. xiangfangensis* was determined by Psortb [26]. Psortb is a web-based tool for pinpointing a protein’s subcellular location, including whether it is periplasmic, cytoplasmic membrane, or cytoplasmic.

### 2.4. Identification of Virulent Proteins

All cytoplasmic proteins were tested for virulence using the virulence factor database (VFDB), which determines the pathogenic virulence of the target proteins [27]. These proteins were considered to be virulence-inducing when they met the following criteria: bit score of more than 100 and sequence identity of more than 30%.

### 2.5. Druggability Analysis and Drug Target Prioritization

Druggability testing was performed on selected virulent proteins. DrugBank is a helpful resource for tracking proteins that are affected by inhibitors and drugs employing a BLAST analysis with an e-value of 10^−5^ [28]. Several factors are used to determine potential therapeutics, including transmembrane helix, molecular weight, stability, molecular functions, and biological processes. TMHMM-2.0 was used to perform transmembrane helix analysis [19]. Since 0 transmembrane helix proteins are easy to express and clone, they were chosen for future research. ProtParam tool was used to calculate the molecular weight (MW) and stability [29,30]. Proteins with stable physicochemical properties and MW < 100 kDa are thought to be the best therapeutic targets. Molecular functions and biological processes were predicted by InterProScan server [31].

### 2.6. Structure Prediction and Preparation

The 3D structure of all target proteins was predicted through I-TASSER server [32]. To evaluate how accurately the model predicts, I-TASSER offers confidence scores. The ProsAweb [33], Verify 3D [34], RAMPAGE [13], and ERRAT [35] tools were further used to validate the quality of all 3D structures. RAMPAGE, which conducts Ramachandran plot analysis, provides a 3D structural validity score for the target proteins. A score of ≥80 was regarded as satisfactory. For further confirmation, ERRAT, an online program that offers details about the protein structure with problematic areas, was used. The quality factor ≥37% was regarded as good.

Predicted 3D structures were prepared for docking using the Molecular Operating Environment (MOE) [36]. Along with the careful algorithm, this tool is quite durable. It also predicts the root mean square deviation (RMSD) and computed energies of docked molecules in addition to the top-ranking positions. These three-dimensional structures underwent 3D protonation and energy minimization, after which they served as templates for molecular docking.

### 2.7. Ligands Retrieval

A total of 2500 plant compounds were retrieved from Pubchem, Zinc database, MAPS, and MPD3 database [37,38]. The partial charges of these compounds were then computed and their energies were minimized via an algorithm for energy minimization with default parameters. The .mdb file was used to store all minimized structures. These ready-made ligands were then utilized as input data for molecular docking.

### 2.8. Molecular Docking and MD Simulation

The molecular docking in the MOE tool was then performed on the minimized structures of the ligands and targeted proteins [37]. After docking, we looked at the best poses for hydrogen bonding/interactions and calculated RMSD in MOE. Chimera was used to investigate the best dock molecules’ orientation [38]. MD simulation is critical for determining how docked complexes keep their structural stability and dynamics. MD simulations of antibacterial drugs bound to target proteins were performed using the AMBER18 software [39]. To create a neutral system, H_2_O molecules were first used to dissolve the top docked complexes, and then counter ions were added. The complexes were then enclosed in a water box that was generated using the TIP3P solvent model and had a thickness of 12 Å [40]. Periodic boundary conditions were used to model the docked complexes. Furthermore, a boundary value of 8 Å was assigned for nonbounded interactions. After 500 cycles of minimizing water molecules, the complete system was reduced to 1000 cycles. Then, each system’s temperature was slowly raised to 300 K. Using the NPT ensemble, the systems were balanced for 100 ps. During the equilibration of counter ions and water molecules, solutes in the first phase were restrained for 50 ps, and protein side chains were then permitted to relax. An MD simulation lasting 100 ns at 300 K and 1 atm was carried out using the NPT ensemble. The SHAKE algorithm was used to manage the covalent and hydrogen bonds [41], and Langevin dynamics were used to control the system’s temperature [42]. AMBER’s CPPTRAJ was used to generate an RMSD plot to confirm that the MD simulation was converging. The initial structure was used as a baseline [43,44,45]. The ligand RMSD method was used to determine the structural flexibility of ligands [46]. The complex’s three-dimensional packing and compactness were investigated in RoG. The average root mean square distance between the average geometric position of an atom and the average position of that atom in a given dynamic is measured by the RMSF [47].

### 2.9. Binding Free Energy Calculation

The MM-GBSA method in AMBER 18 was used to calculate the binding free energies (ΔGtol) of E. xiangfangensis proteins complexed with the most promising hit compounds. In short, 10,000 snapshots were made from the last 20 ns of stable paths for each system, with a 2 ps gap between each one. The sum of the molecular mechanics binding energy (EMM) and the solvation free energy (Gsol) equals the total binding free energy, as illustrated below.

ΔEgas = ΔEele + ΔEint + ΔEvdw

ΔGsol = ΔGp + ΔGNp

ΔGtol = ΔEMM + ΔGsol

where EMM is subdivided further into electrostatic energy (ΔEele), van der Waals energy (ΔEvdw), and internal energy (ΔEint). The sum of the polar (ΔGp) and nonpolar (ΔGNp) components determines the total solvation free energy (ΔGsol). The MM-GBSA method has been proven to be accurate in the assessment of binding-free antibacterial inhibitors.

### 2.10. Physiochemical Profiling

Drug-likeness and molecular descriptors of phytochemicals with the highest docking scores were examined through the Molinspiration server, which makes predictions based on the “rule of five” [48,49]. Criteria include having a molecular mass less than 500 Daltons, an analogue P value less than 5, 5 hydrogen bond donors, and up to 10 acceptors of hydrogen bonds. AdmetSAR can be used to analyze the pharmacokinetic characteristics of substances, including their distribution, metabolism, adsorption, toxicity, and excretion [50].

## 3. Results

### 3.1. E. xiangfangensis Proteome Retrieval and Identification of Essential Nonhomologous Proteins

In this study, six complete proteomes of *E. xiangfangensis* were obtained from the NCBI database: (i) GCA_003999755.1, (ii) GCA_000814225.1, (iii) GCA_001729785.1, (iv) GCA_003964795.2, (v) GCA_014931695.1, and (vi) GCA_000807405.4). Several filters were used in the retrieval step, including complete proteomes, humans as hosts, and incomplete proteomes. Core proteome of *E. xiangfangensis* was extracted from the six complete proteomes using the OrthoFinder program. The pathogen has 21,720 core proteins, according to the OrthoFinder results, while the CD-HIT analysis found that there are 19,109 redundant proteins and 2611 nonredundant proteins in the pathogen core proteins. The essential *E. xiangfangensis* proteins were predicted using Geptop2.0 (Chengdu, China). Out of 2611 nonredundant proteins, 372 were obtained from Geptop2.0 as essential proteins. Nonhomologous analysis was used to find protein targets that are absent from the host (Homo sapiens) in the 372 essential proteins. *E. xiangfangensis* proteins and the proteome of Homo sapiens were compared. Only 195 proteins were discovered to be similar to human proteins as a result of this analysis, with the remaining 177 proteins being classified as nonhomologous due to their lack of significant resemblance.

### 3.2. Subcellular Localization

Proteins can be identified as vaccine or drug targets based on their localization. The 177 proteins chosen for this study were also examined for subcellular localization. According to the findings, 133 of the 177 proteins were located in the cytoplasm. The 133 cytoplasmic proteins were added for further examination because they can be used as drug targets.

### 3.3. Identification of Virulent Proteins and Druggability Analysis

All cytoplasmic proteins were examined using BLASTp against the virulence factor database, which classifies 20 therapeutic targets as virulent. Another crucial characteristic for possible therapeutic targets is druggability. Druggable targets are proteins that have already been targeted by drugs, while novel targets are proteins that have yet to be targeted. According to the findings, seven proteins did not match any of the DrugBank drug targets, while the remaining thirteen proteins did. These seven proteins were, therefore, viewed as novel targets and became the topic of additional research.

### 3.4. Drug Target Prioritization

Several factors were taken into consideration to identify the potential therapeutic targets. Transmembrane helices of novel drug targets were predicted by TMHMM server. Proteins having 0 transmembrane helices are considered as good drug targets. Out of seven novel targets, six proteins were observed to have 0 transmembrane helices. Molecular weight and stability of these six proteins were checked by Protaparam server. Out of six, four proteins were found to be stable and have MW < 100 kDa. Drug discovery depends on understanding the biological process, molecular function, and structural information of proteins. According to functional prediction results of InterProScan, three proteins were screened as drug targets. Details of these three drug targets are listed in Table 1.

### 3.5. Structure Prediction

**Figure 1:**
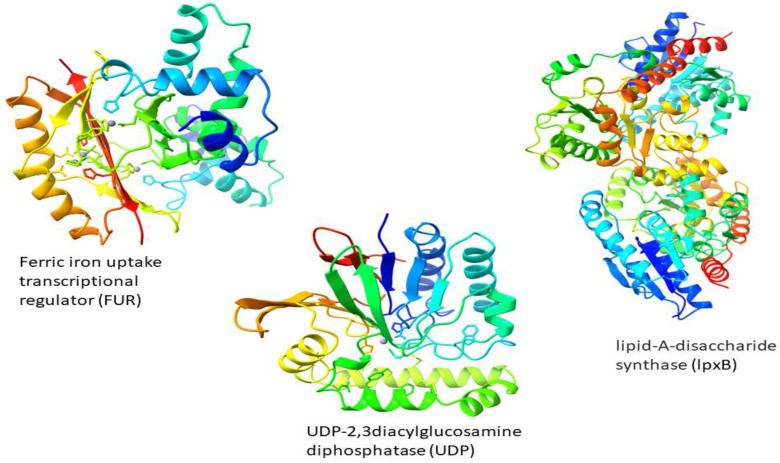
3D structures of drug targets predicted by I-Tasser.

3D structures of three target proteins were predicted by I-Tasser (Figure 1). ProsAweb, Verify 3D, RAMPAGE, and ERRAT were used to validate the quality factor of 3D structures of target proteins. As shown in Table 2, the quality factors/compatibility score predicted by tools were ≥80. These scores indicate that our proteins’ 3D structures are suitable for docking.

### 3.6. Molecular Docking

**Figure 2:**
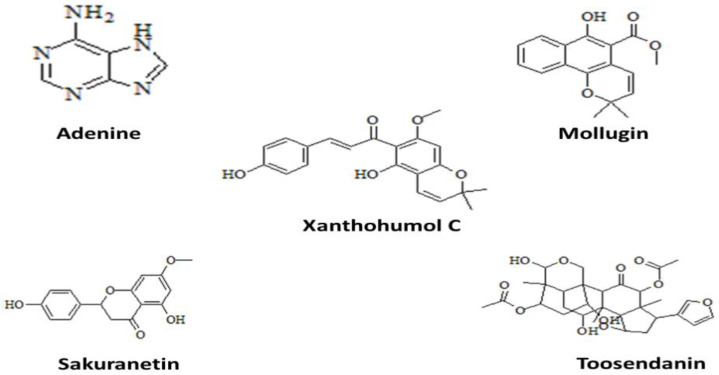
2D structures of top five plant compounds.

Docking against three drug targets with 2500 plant compounds was carried out using the MOE tool. After redocking the top 100 compounds into target protein binding pockets, the top five molecules were selected. The interaction residues (Table 3) of all three target proteins were found to bind with great affinity to adenine, Mollugin, Xanthohumol C, Sakuranetin, and Toosendanin (Figure 2).

**Figure 3:**
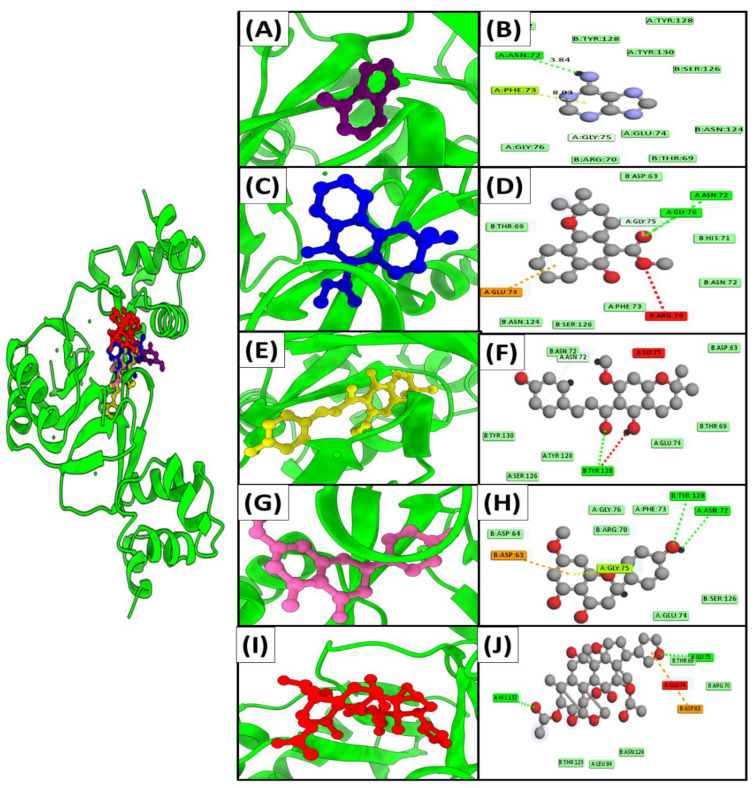
Interaction mechanisms and binding modes of novel FUR protein inhibitors. A 3D close view into the binding mode of (**A**) Adenine, (**C**) Mollugin, (**E**) Xanthohumol C, (**G**) Sakuranetin, and (**I**) Toosendanin. 2D interaction analysis of (**B**) Adenine, (**D**) Mollugin, (**F**) Xanthohumol C, (**H**) Sakuranetin, and (**J**) Toosendanin.

Adenine bound to FUR protein with a binding score of −18.7 kcal/mol by creating hydrogen bonds with the side chains of Asn A72,Phe A73,Gly A75,Glu A74, whereas Mollugin is bound with a binding value of −16.2 kcal/mol by making hydrogen bonds with Glu A74,Arg B70,Gly A76,Asn A72,Gly A75. Adenine and Mollugin bind strongly to FUR active residues, followed by Xanthohumol C, Sakuranetin, and Toosendanin, which have binding scores of −14.5 kcal/mol, −13.6 kcal/mol, and −13.1 kcal/mol, respectively (Table 3). All ligands, excluding Sakuranetin, created strong hydrogen bonds with the conserved Gly A75 (Figure 3).

**Figure 4:**
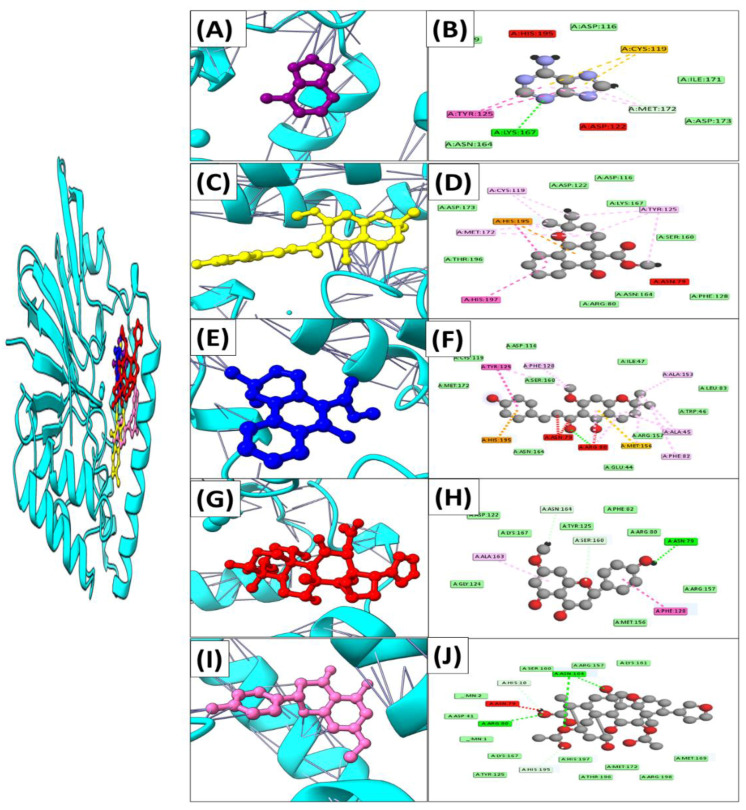
Interaction mechanisms and binding modes of novel UDP protein inhibitors. A 3D close view into the binding mode of (**A**) Adenine, (**C**) Mollugin, (**E**) Xanthohumol C, (**G**) Sakuranetin, and (**I**) Toosendanin. 2D interaction analysis of (**B**) Adenine, (**D**) Mollugin, (**F**) Xanthohumol C, (**H**) Sakuranetin, and (**J**) Toosendanin.

Similarly, Adenine, Mollugin, Xanthohumol C, Sakuranetin, and Toosendanin have been found to bind through significant hydrogen bonds in UDP protein, with binding scores of −11.6 kcal/mol, −19.8 kcal/mol, −15.3 kcal/mol, −14.9 kcal/mol, and −17.6 kcal/mol, respectively. As illustrated in Table 3 and Figure 4, all of the active site’s critical residues function as electron donors in the development of a H-bond network.

**Figure 5:**
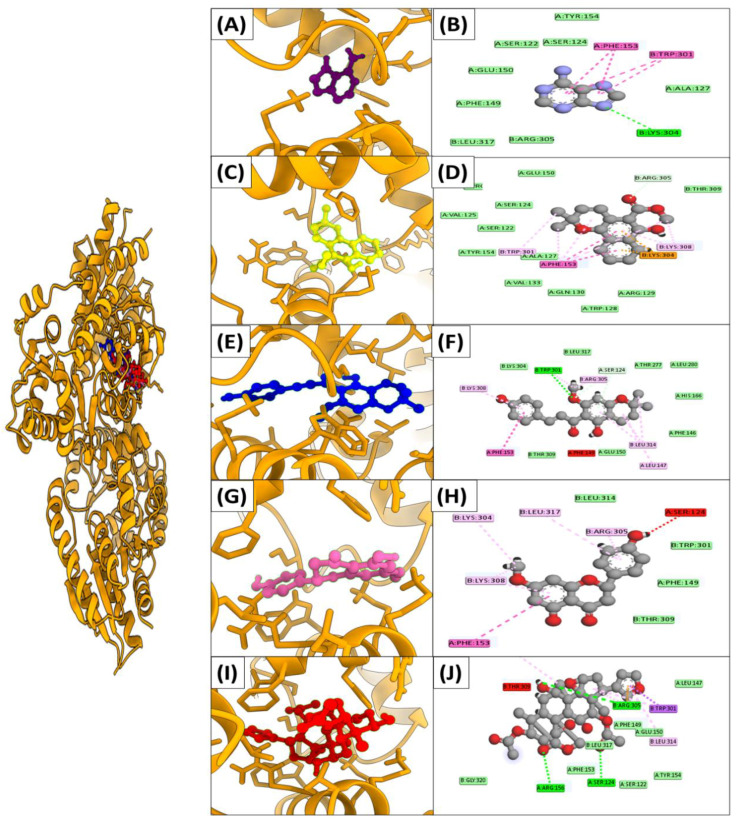
Interaction mechanisms and binding modes of novel **lpxB** protein inhibitors. A 3D close view into the binding mode of (**A**) Adenine, (**C**) Mollugin, (**E**) Xanthohumol C, (**G**) Sakuranetin, and (**I**) Toosendanin. 2D interaction analysis of (**B**) Adenine, (**D**) Mollugin, (**F**) Xanthohumol C, (**H**) Sakuranetin, and (**J**) Toosendanin.

Similarly, the top five inhibitors (Adenine, Mollugin, Xanthohumol C, Sakuranetin, and Toosendanin) found to inhibit FUR and UDP proteins were also found to inhibit lpxB protein. The binding energies of the five active compounds ranged from −14.3 to −19.3 kcal/mol. Most compounds formed hydrogen bonds with Lys B304, Trp B301, and Phe A153, indicating that these compounds may play a role in disease management. Hydrogen interactions between the lpxB protein residues’ side chains and backbone atoms stabilized the inhibitors spatially within the pocket (Figure 5).

All of the top five inhibitors formed strong bonds with functionally and structurally important interacting sites of the *E. xiangfangensis* proteins. The compounds identified in this study may have additive or synergistic anti-*E. xiangfangensis* effects.

### 3.7. MD Simulation

To obtain a deeper comprehension of the dynamics of targets in the presence of screened hits, a 100-ns molecular dynamic simulation was performed. Statistical indicators, such as RMSD, RMSF, and radius of gyration were used to confirm the structural stability of docked complexes. The root mean square deviations (RMSD) of carbon alpha atoms were examined first.

### 3.8. Root Mean Square Deviations (RMSD)

**Figure 6:**
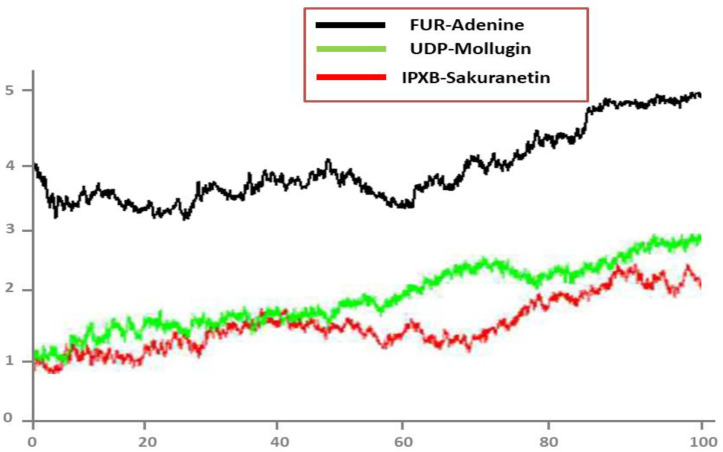
RMSD analysis of top docked complexes.

The RMSD value deviates from the intermolecular conformation of the initially docked complex, indicating structural modifications [49]. A uniform RMSD plot reveals increased intermolecular strength and system structural equilibrium as simulation time progresses (Figure 6). During the first 50 ns of simulation time, the Adenine/FUR complex exhibited stability. Following this, the complex exhibited a slight deviation of 0.5 Ao for the next 85 ns, after which it remained stable (Figure 6). The second complex (Mollugin/UDP) showed minor deviations on its first jump and achieved stability, as illustrated in green color in Figure 6. The third complex (Sakuranetin/IPXB) exhibited a modest deviation of 0.3 Ao between 55 and 70 ns but otherwise remained stable, as shown by the red line (Figure 6).

### 3.9. Root Mean Square Fluctuations (RMSF)

**Figure 7:**
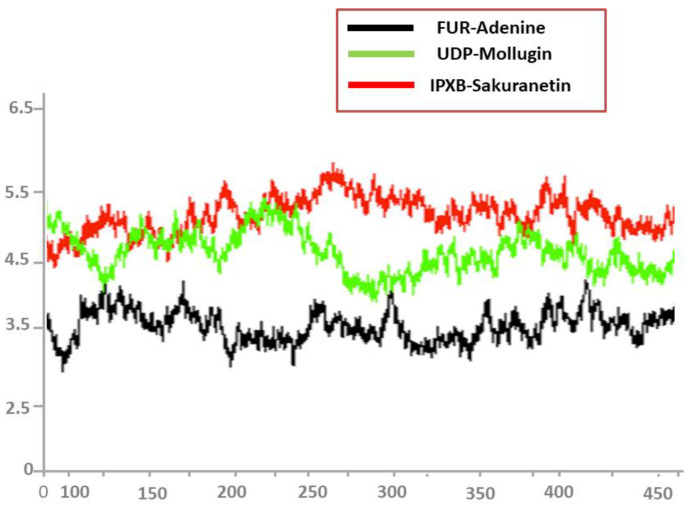
RMSF analysis of top docked complexes.

After that, the root mean square fluctuations (RMSF) of the simulated complexes were computed. RMSF analysis facilitates the identification of flexible residues in particular proteins and the comprehension of how these differences affect the stability of complexes (Figure 7). Graphs of the Adenine/FUR complex indicate minor fluctuations and showed overall stability up to residue number 450, while second Mollugin/UDP complex and third Sakuranetin/IPXB complex showed a deviation between the residue numbers 325 to 425, as indicated in red and green color, respectively, in Figure 7.

### 3.10. Radius of Gyration (RoG) 

**Figure 8:**
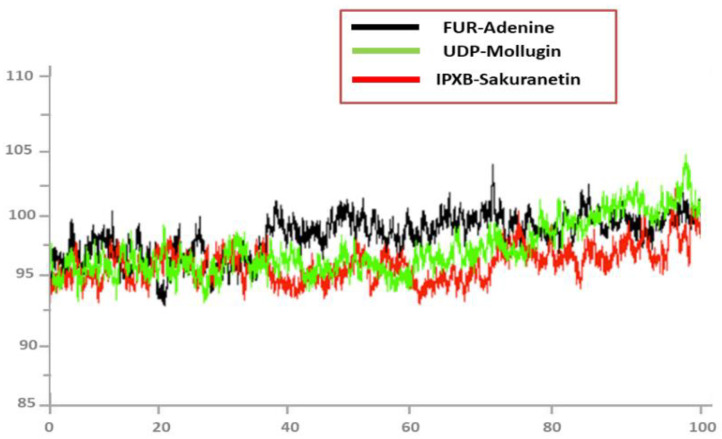
RoG analysis of top docked complexes.

During the simulation, Rg analysis was used to measure structural equilibrium and protein density. The best Rg value for globular proteins should be low, but the best Rg value for protein forms with more turns and loops could be much higher, as shown in Figure 8. Rog values of the complexes are follows: Adenine/FUR complex (maximum, 95.45 Å; mean, 95.12 Å), Mollugin/UDP (maximum, 99.82 Å; mean, 95.12 Å), and Sakuranetin/IPXB (maximum, 96.03 Å; mean, 94.25 Å). During the simulation period, no notable reduction in compactness was detected in any of the complexes.

### 3.11. Binding Free Energy Calculations

MMGBSA/MMPBSA methods were used to estimate binding free energies to learn more about how well the complexes bind to *E. xiangfangensis* proteins. Stable complexes are made because all the binding interactions are energetically good. In all complexes, gas-phase energy predominates the system energy, with van der Waals playing a significant part and electrostatic energy playing a minor role. The polar solvation energy is shown to be unfavorable in binding, but the nonpolar energy appears to be advantageous in complex equilibration. Table 4 lists the complexes’ binding energies in detail.

### 3.12. Drug Scan/ADMET 

Based on Lipinski’s Rules of Five, Molinspiration predicted the drug-likeness of five compounds. The selected candidates did not violate the “rule of five” and displayed drug-like qualities in Table 5. The admetSAR server was used to examine the pharmacokinetic properties of all of the candidate compounds, and the findings are shown in Table 6.

## 4. Discussion

*Enterobacter xiangfangensis* is a new bacterial pathogen from the Enterobacter genus that can become resistant to many antibiotics. To deal with this potentially fatal situation, it is urgent that drugs to treat *E. xiangfangensis* should be developed. The identification of therapeutic targets is a vital step in computer-aided drug design methods [51]. Recent advances in computational biology and bioinformatics have produced a variety of methods for in silico analysis and drug design, which has reduced the time and cost of trial and error in the drug development process [7].

The democratization of sequencing has made it simpler to generate genomic sequence data, so multiple or pan genome analyses are being used to identify key therapeutic proteins in the bacterial species, making the therapeutic candidates universal. This replaces the practice of using a single genome as a reference [52]. To obtain accurate gene information and take into consideration genetic variation within species, a pan-genomics-mediated technique was used in this study. The current study screened for potential novel putative therapeutic targets in *E. xiangfangensis* using a pan genome and subtractive genomics strategy. The potential therapeutic targets of several bacteria, including *Stenotrophomonas maltophilia* [53], *Mycobacterium tuberculosis* [54], and *Streptococcus gallolyticus* [55] have also been predicted using these methods. Although, some additional analyses have been performed in our study that make it innovative from other studies, such as prediction of molecular function and biological processes, transmembrane helices, and druggability analysis.

In this study, six fully sequenced proteomes of *E. xiangfangensis* were downloaded from NCBI and their pan genome analysis was performed. The core proteome, which contains 21,720 proteins, was evaluated with CD-HIT to identify any duplication, resulting in a total of 2611 nonredundant proteins. The analysis of essential proteins is critical for the development of antipathogen drugs. Essential proteins are required for the pathogen’s growth, survival, adaptability, and replication. The same function is carried out by these proteins in several organisms, and they have evolutionary relationships with other proteins. Pathogens may die if essential proteins are targeted. Hence, 372 essential proteins were identified among nonredundant proteins. Shahid et al. discovered 394 essential proteins in *Shigella sonnei*, Mehmood et al. discovered 208 essential proteins in *Mycoplasma pneumoniae*, and Rehman et al. discovered 302 essential proteins in *Streptococcus Pyogenes* using this method [7,56,57]. These genes could be related to humans. Thus, targeting such genes can disrupt human metabolism and be fatal. Cross-reactivity and adverse events can be avoided by selecting nonhomologous proteins that are not present in *homo sapiens*. So, 177 nonhomologous proteins were screened to prevent such unfavorable conditions and toxicity. Virulent factors aid bacteria in evading host defenses and contribute to pathogenicity, making them suitable therapeutic targets. A total of 20 virulent proteins were identified from 177 nonhomologous proteins. Protein localization is closely related to biological function, making it crucial to predict where proteins will be found within cells. Proteins can typically be found in five major locations: the outer membrane, the plasma membrane, the extracellular membrane, the periplasm, and the cytoplasm. Protein localization can be used to assess whether a protein is a drug or vaccine target; cytoplasmic proteins are therapeutic targets. Hence, three cytoplasmic proteins: ferric iron uptake transcriptional regulator (FUR), UDP-2,3diacylglucosamine diphosphatase (UDP), and lipid-A-disaccharide synthase (lpxB) were identified as drug targets on the basis of several factors, including druggability, transmembrane helices, molecular weight, stability, and molecular and biological function. Tertiary structures of target proteins were predicted, assessed, and verified.

Molecular docking has become a lightning rod for validating the stability between compounds and targets [58,59,60]. Molecular docking approach was employed to identify the compounds with the best residue interactions with the target proteins. Out of 5000 docked molecules, 5 molecules that interact with all three proteins were chosen: Adenine, Mollugin, Xanthohumol C, Sakuranetin, and Toosendanin, based on a low score and a high number of interacting residues. Drug probability and the molecular profile of these five compounds were evaluated using “Lipinski’s Rule of Five”. They all followed “Lipinski’s Rule of Five”.

Afterwards, the compounds were tested for human intestine absorption (HIA), BBB penetration, and AMES monitoring. By analyzing the ADMET features, it is possible to anticipate the toxicity level, behavior, and outcome of a drug candidate in the human body [61]. A candidate’s likelihood of passing across the blood–brain barrier, metabolism, subcellular localization, intestinal absorption, and—most notably—degree of harm it can inflict on the body are all provided by this test [62]. These compounds have no deleterious absorption effects., according to their ADMET profiles. Additionally, when compared to the AMES test, none of the compounds displayed any toxicity or mutagenic effects.

Hence, the novel drug targets identified in this study may be highly valuable in the drug therapeutic field for designing new formulations of drug molecules and discovering inhibitors to control *E. xiangfangensis* function, although further experimental research is still required to validate these drug targets.

## 5. Conclusions

The novel bacteria *Enterobacter xiangfangensis* is susceptible to developing drug resistance. Therefore, it is critical that drugs should be developed to treat *E. xiangfangensis*. The current study used pan genome analysis to discover 21,720 key proteins from six *E. xiangfangensis* strains using an in-silico technique. Twenty targets were ultimately chosen after subtractive genomics and the identification of essential genes. Utilizing 3D structural information and drug prioritization, three possible therapeutic targets were prioritized among these proteins. Additionally, active molecules were found using molecular docking analysis, and the top five active molecules were picked based on the number of interactions, binding free energy, and drug score. The discovered novel drug targets might have advanced to the early phases of the drug design phase for the potential screening of new therapeutic candidates and are, consequently, suggested as an antibacterial therapy. Drug target experimental evaluation and subsequent drug molecule design against any target is a time-demanding and expensive job. Hence, the findings of our study will substantially aid the therapeutic development process against *E. xiangfangensis*. However, computational analyses have limits; hence, more in vitro and in vivo investigations to evaluate the inhibitory ability of chosen promising candidates against *E. xiangfangensis* are necessary.

## Figures and Tables

**Table 1:** Details of selected drug targets.

Proteins	Subcellular Localization	Transmembrane Helices	Molecular Weight	Stability	Molecular Function	Biological Processes
Ferric iron uptake transcriptional regulator (FUR)	Cytoplasm	0	16,765.81	Stable	DNA-binding transcription factor activity	regulation of transcription, DNA-templated
UDP-2,3diacylglucosamine diphosphatase (UDP)	Cytoplasm	0	26,832.02	Stable	pyrophosphatase activity hydrolase activity	lipid A biosynthetic process
lipid-A-disaccharide synthase (lpxB)	Cytoplasm	0	42,472.56	Stable	lipid-A-disaccharide synthase activity	lipid A biosynthetic process

**Table 2:** Structural validation of target proteins.

Scores	FUR Protein	UDP Protein	lpxB Protein
C-score	−6.02	−4.98	−7.87
Estimated TM-score	0.91 ± 0.05	0.85 ± 0.09	0.74 ± 0.08
**ProSA**			
Z Score	−7.65	−8.35	−6.98
**Verify 3D**			
Compatibility Score	81.71	83.89	80.03
**Errat**			
Quality Factor	91.76	87.56	90.67
**Ramachandran plot (%)**			
Core	90.2%	83.7%	88.7%
Allowed	6.6%	12.8%	7.9%
General	2.0%	1.4%	2.9%
Disallowed	1.9%	1.5%	1.8%

**Table 3:** Docking statistics of target proteins against plant compounds.

Compounds Name and ID	FUR Protein				UDP Protein				lpxB Protein			
	Binding Affinity	Inhibition Constant	RMSD	Interacting Residues	Binding Affinity	Inhibition Constant	RMSD	Interacting Residues	Binding Affinity	Inhibition Constant	RMSD	Interacting Residues
Adenine (190)	−18.7	67.1 μM	0.9	Asn A72,Phe A73,Gly A75,Glu A74	−11.6	58.7 μM	2.5	Cys A119,His A195,Tyr A125,Lys A167,Asp A122,Met A172	−15.3	69.9 μM	1.8	Phe A153,Trp B301,Lys B304
Mollugin (124219)	−16.2	72.2 μM	1.2	Glu A74,Arg B70,Gly A76,Asn A72,Gly A75	−19.8	75.2 μM	0.7	Tyr A125,Cys A119,Met A172,His A197	−18.8	89.6 μM	1.1	Trp B301,Phe A153,Lys B 304
xanthohumol C (10338075)	−14.5	85.2 μM	1.8	Tyr B128,Asn B72,Gly A75,	−15.3	80.1 μM	1.5	Ala A153,Ala A45,Met A156	−16.2	72.7 μM	0.8	Leu A147,Leu B314,Phe A153,Trp B301
Sakuranetin (73571)	−13.6	76.4 μM	0.8	Tyr B128,Asn A72,Asp B63	−14.9	90.3 μM	2.1	Ser A160,Asn A79,Phe A 128,Ala A163,Asn A164	−19.3	80.2 μM	2.3	Phe A153,Lys B304,Leu B317,Lys B308
Toosendanin (115060)	−13.1	93.1 μM	2.0	His A132,Thr B69,Gly A75,GluA74,Asp B63	−17.6	63.9 μM	0.9	Asn A164,Arg A80,Asn A79,His A10	−14.3	70.4 μM	2.9	Lys B308,Arg A156,Ser A124,Trp B301

**Table 4:** Binding energies of best docked compounds.

Energy Component	Adenine	Mollugin	Xanthohumol C	Sakuranetin	Toosendanin
Van der Waals	−45.61	−34.06	−39.19	−42.12	−44.71
Electrostatic	−41.95	−26.23	−37.69	−34.69	−33.96
Polar solvation	59.79	45.10	52.45	55.02	65.08
Nonpolar solvation	−4.40	−6.90	−5.32	−4.49	−7.70
Net gas phase	−78.23	−70.79	−61.12	−45.05	−59.45
Net solvation	60.28	55.17	46.41	61.77	45.31
Net complex energy	−35.52	−50.18	−45.41	−42.21	−50.45

**Table 5:** Drug-likeness properties of potential compounds.

Ligands	Molecular Weight	Molecular Formula	Hydrogen Bond Donor	Hydrogen Bond Acceptor	XLogP3	Heavy Atom Count
Adenine	135.13	C_5_H_5_N_5_	2	4	−0.1	10
Mollugin	284.31	C_17_H_16_O_4_	1	4	4.1	21
xanthohumol C	352.4	C_21_H_20_O_5_	2	5	4.4	26
Sakuranetin	286.28	C_16_H_14_O_5_	2	5	2.7	21
Toosendanin	574.6	C_30_H_38_O_11_	3	10	0.7	41

**Table 6:** ADMET properties of the finest docked compounds.

Compounds	Adenine	Mollugin	Xanthohumol C	Sakuranetin	Toosendanin
**Absorption/Distribution**					
Blood–Brain Barrier	No	No	No	No	No
Log *S*	−410	−3.70	−4.12	−4.76	−4.94
GI Absorption	High	Low	High	High	Low
Caco-2 permeability	−5.18	−8.98	−6.71	−6.54	−7.72
Bioavailability Score	0.55	0.55	0.55	0.55	0.17
**Metabolism**					
P-gp substrate	No	No	Yes	No	No
CYP1A2 inhibitor	No	Yes	No	Yes	Yes
CYP2C19 inhibitor	No	No	Yes	Yes	Yes
CYP2C9 inhibitor	No	Yes	No	No	Yes
CYP2D6 inhibitor	No	Yes	Yes	No	No
CYP3A4 inhibitor	No	Yes	Yes	Yes	Yes
**Toxicity**					
AMES Toxicity	Nill	Nill	Nill	Nill	Nill
Carcinogenicity	None	None	None	None	None
Immunogenicity	NT	NT	NT	NT	NT
Acute Oral Toxicity	NT	NT	NT	NT	NT

NT: Nontoxic.

## References

[1] Ramirez D., Giron M. (2021). Enterobacter infections. StatPearls.

[2] Davin-Regli A., Pagès J.-M. (2015). *Enterobacter* aerogenes and *Enterobacter* cloacae; versatile bacterial pathogens confronting antibiotic treatment. Front. Microbiol..

[3] Sanders W.E., Sanders C.C. (1997). *Enterobacter* spp.: Pathogens poised to flourish at the turn of the century. Clin. Microbiol. Rev..

[4] Przedborski S., Vila M., Jackson-Lewis V. (2003). Series Introduction: Neurodegeneration: What is it and where are we?. J. Clin. Investig..

[5] Lamptey R.N., Chaulagain B., Trivedi R., Gothwal A., Layek B., Singh J. (2022). A Review of the Common Neurodegenerative Disorders: Current Therapeutic Approaches and the Potential Role of Nanotherapeutics. Int. J. Mol. Sci..

[6] Ziukelis E.T., Mak E., Dounavi M.-E., Su L., O’Brien J. (2022). Fractal dimension of the brain in neurodegenerative disease and dementia: A systematic review. Ageing Res. Rev..

[7] Du X., Wang X., Geng M. (2018). Alzheimer’s disease hypothesis and related therapies. Transl. Neurodegener..

[8] Scorza F.A., Guimarães-Marques M., Nejm M., de Almeida A.C.G., Scorza C.A., Fiorini A.C., Finsterer J. (2022). Sudden unexpected death in Parkinson’s disease: Insights from clinical practice. Clinics.

[9] Basharat Z., Jahanzaib M., Rahman N. (2021). Therapeutic target identification via differential genome analysis of antibiotic resistant Shigella sonnei and inhibitor evaluation against a selected drug target. Infect. Genet. Evol..

[10] Muddapu V.R., Dharshini S.A.P., Chakravarthy V.S., Gromiha M.M. (2020). Neurodegenerative diseases–is metabolic deficiency the root cause?. Front. Neurosci..

[11] Aslam S., Mehmood M.A., Rahman M.-u., Noor F., Ahmad N. (2022). Bioinformatics-assisted multiomics approaches to improve the agronomic traits in cotton. Bioinformatics in Agriculture.

[12] Noor F., Ahmad S., Saleem M., Alshaya H., Qasim M., Rehman A., Ehsan H., Talib N., Saleem H., Bin Jardan Y.A. (2022). Designing a multi-epitope vaccine against Chlamydia pneumoniae by integrating the core proteomics, subtractive proteomics and reverse vaccinology-based immunoinformatics approaches. Comput. Biol. Med..

[13] Noor F., Ashfaq U.A., Javed M.R., Saleem M.H., Ahmad A., Aslam M.F., Aslam S. (2021). Comprehensive computational analysis reveals human respiratory syncytial virus encoded microRNA and host specific target genes associated with antiviral immune responses and protein binding. J. King Saud Univ.-Sci..

[14] Noor F., Noor A., Ishaq A.R., Farzeen I., Saleem M.H., Ghaffar K., Aslam M.F., Aslam S., Chen J.-T. (2021). Recent advances in diagnostic and therapeutic approaches for breast cancer: A comprehensive review. Curr. Pharm. Des..

[15] Choonara Y.E., Pillay V., Du Toit L.C., Modi G., Naidoo D., Ndesendo V.M., Sibambo S.R. (2009). Trends in the molecular pathogenesis and clinical therapeutics of common neurodegenerative disorders. Int. J. Mol. Sci..

[16] Kiaei M. (2013). New hopes and challenges for treatment of neurodegenerative disorders: Great opportunities for young neuroscientists. Basic Clin. Neurosci..

[17] Almatroudi A. (2022). Non-Coding RNAs in Tuberculosis Epidemiology: Platforms and Approaches for Investigating the Genome’s Dark Matter. Int. J. Mol. Sci..

[18] Das T., Das T.K., Khodarkovskaya A., Dash S. (2021). Non-coding RNAs and their bioengineering applications for neurological diseases. Bioengineered.

[19] Qamar M.T.U., Zhu X., Khan M.S., Xing F., Chen L.-L. (2020). Pan-genome: A promising resource for noncoding RNA discovery in plants. Plant Genome.

[20] Koch A., Cox H., Mizrahi V. (2018). Drug-resistant tuberculosis: Challenges and opportunities for diagnosis and treatment. Curr. Opin. Pharmacol..

[21] Qureshi I.A., Mehler M.F. (2012). Emerging roles of non-coding RNAs in brain evolution, development, plasticity and disease. Nat. Rev. Neurosci..

[22] Rehman A., Wang X., Ahmad S., Shahid F., Aslam S., Ashfaq U.A., Alrumaihi F., Qasim M., Hashem A., Al-Hazzani A.A. (2021). In Silico Core Proteomics and Molecular Docking Approaches for the Identification of Novel Inhibitors against Streptococcus pyogenes. Int. J. Environ. Res. Public Health.

[23] Salvatori B., Biscarini S., Morlando M. (2020). Non-coding RNAs in nervous system development and disease. Front. Cell Dev. Biol..

[24] Dahariya S., Paddibhatla I., Kumar S., Raghuwanshi S., Pallepati A., Gutti R.K. (2019). Long non-coding RNA: Classification, biogenesis and functions in blood cells. Mol. Immunol..

[25] Slaby O., Calin G.A. (2016). Non-Coding RNAs in Colorectal Cancer.

[26] Nelson W.C., Stegen J.C. (2015). The reduced genomes of Parcubacteria (OD1) contain signatures of a symbiotic lifestyle. Front. Microbiol..

[27] Memon D., Bi J., Miller C.J. (2019). In silico prediction of housekeeping long intergenic non-coding RNAs reveals HKlincR1 as an essential player in lung cancer cell survival. Sci. Rep..

[28] Sun J., Lin Y., Wu J. (2013). Long non-coding RNA expression profiling of mouse testis during postnatal development. PLoS ONE.

[29] Guglas K., Bogaczyńska M., Kolenda T., Ryś M., Teresiak A., Bliźniak R., Łasińska I., Mackiewicz J., Lamperska K. (2017). lncRNA in HNSCC: Challenges and potential. Contemp. Oncol. Współczesna Onkol..

[30] Kuhn D.E., Martin M.M., Feldman D.S., Terry A.V., Nuovo G.J., Elton T.S. (2008). Experimental validation of miRNA targets. Methods.

[31] Bavelloni A., Ramazzotti G., Poli A., Piazzi M., Focaccia E., Blalock W., Faenza I. (2017). MiRNA-210: A current overview. Anticancer Res..

[32] Noor F., Saleem M.H., Aslam M.F., Ahmad A., Aslam S. (2021). Construction of miRNA-mRNA network for the identification of key biological markers and their associated pathways in IgA nephropathy by employing the integrated bioinformatics analysis. Saudi J. Biol. Sci..

[33] Sufyan M., Ashfaq U.A., Ahmad S., Noor F., Saleem M.H., Aslam M.F., El-Serehy H.A., Aslam S. (2021). Identifying key genes and screening therapeutic agents associated with diabetes mellitus and HCV-related hepatocellular carcinoma by bioinformatics analysis. Saudi J. Biol. Sci..

[34] Noor F., Saleem M.H., Javed M.R., Chen J.-T., Ashfaq U.A., Okla M.K., Abdel-Maksoud M.A., Alwasel Y.A., Al-Qahtani W.H., Alshaya H. (2022). Comprehensive computational analysis reveals H5N1 influenza virus-encoded miRNAs and host-specific targets associated with antiviral immune responses and protein binding. PLoS ONE.

[35] Gan E.-S., Huang J., Ito T. (2013). Functional roles of histone modification, chromatin remodeling and microRNAs in Arabidopsis flower development. Int. Rev. Cell Mol. Biol..

[36] Beermann J., Piccoli M.-T., Viereck J., Thum T. (2016). Non-coding RNAs in development and disease: Background, mechanisms, and therapeutic approaches. Physiol. Rev..

[37] Winter J., Jung S., Keller S., Gregory R.I., Diederichs S. (2009). Many roads to maturity: MicroRNA biogenesis pathways and their regulation. Nat. Cell Biol..

[38] Curtis H.J., Sibley C.R., Wood M.J. (2012). Mirtrons, an emerging class of atypical miRNA. Wiley Interdiscip. Rev. RNA.

[39] Juźwik C.A., Drake S.S., Zhang Y., Paradis-Isler N., Sylvester A., Amar-Zifkin A., Douglas C., Morquette B., Moore C.S., Fournier A.E. (2019). microRNA dysregulation in neurodegenerative diseases: A systematic review. Prog. Neurobiol..

[40] Lang M.-F., Shi Y. (2012). Dynamic roles of microRNAs in neurogenesis. Front. Neurosci..

[41] Ponomarev E.D., Veremeyko T., Barteneva N., Krichevsky A.M., Weiner H.L. (2011). MicroRNA-124 promotes microglia quiescence and suppresses EAE by deactivating macrophages via the C/EBP-α–PU. 1 pathway. Nat. Med..

[42] Coolen M., Katz S., Bally-Cuif L. (2013). miR-9: A versatile regulator of neurogenesis. Front. Cell. Neurosci..

[43] Pathania M., Torres-Reveron J., Yan L., Kimura T., Lin T.V., Gordon V., Teng Z.-Q., Zhao X., Fulga T.A., Van Vactor D. (2012). miR-132 enhances dendritic morphogenesis, spine density, synaptic integration, and survival of newborn olfactory bulb neurons. PLoS ONE.

[44] Paraskevopoulou M.D., Georgakilas G., Kostoulas N., Reczko M., Maragkakis M., Dalamagas T.M., Hatzigeorgiou A.G. (2013). DIANA-LncBase: Experimentally verified and computationally predicted microRNA targets on long non-coding RNAs. Nucleic Acids Res..

[45] Chen X., Yan G.-Y. (2013). Novel human lncRNA–disease association inference based on lncRNA expression profiles. Bioinformatics.

[46] Ferre F., Colantoni A., Helmer-Citterich M. (2016). Revealing protein–lncRNA interaction. Brief. Bioinform..

[47] Kawaguchi T., Tanigawa A., Naganuma T., Ohkawa Y., Souquere S., Pierron G., Hirose T. (2015). SWI/SNF chromatin-remodeling complexes function in noncoding RNA-dependent assembly of nuclear bodies. Proc. Natl. Acad. Sci. USA.

[48] Quinodoz S., Guttman M. (2014). Long noncoding RNAs: An emerging link between gene regulation and nuclear organization. Trends Cell Biol..

[49] Ansari M.A., Khan F.B., Safdari H.A., Almatroudi A., Alzohairy M.A., Safdari M., Amirizadeh M., Rehman S., Equbal M.J., Hoque M. (2021). Prospective therapeutic potential of Tanshinone IIA: An updated overview. Pharmacol. Res..

[50] Parasramka M.A., Maji S., Matsuda A., Yan I.K., Patel T. (2016). Long non-coding RNAs as novel targets for therapy in hepatocellular carcinoma. Pharmacol. Ther..

[51] Pamudurti N.R., Bartok O., Jens M., Ashwal-Fluss R., Stottmeister C., Ruhe L., Hanan M., Wyler E., Perez-Hernandez D., Ramberger E. (2017). Translation of circRNAs. Mol. Cell.

[52] Basharat Z., Jahanzaib M., Yasmin A., Khan I.A. (2021). Pan-genomics, drug candidate mining and ADMET profiling of natural product inhibitors screened against Yersinia pseudotuberculosis. Genomics.

[53] Saleem H., Ashfaq U.A., Nadeem H., Zubair M., Siddique M.H., Rasul I. (2021). Subtractive genomics and molecular docking approach to identify drug targets against Stenotrophomonas maltophilia. PLoS ONE.

[54] Dar H.A., Zaheer T., Ullah N., Bakhtiar S.M., Zhang T., Yasir M., Azhar E.I., Ali A. (2020). Pangenome Analysis of Mycobacterium tuberculosis Reveals Core-Drug Targets and Screening of Promising Lead Compounds for Drug Discovery. Antibiotics.

[55] Qureshi N.A., Bakhtiar S.M., Faheem M., Shah M., Bari A., Mahmood H.M., Sohaib M., Mothana R.A., Ullah R., Jamal S.B. (2021). Genome-based drug target identification in human pathogen Streptococcus gallolyticus. Front. Genet..

[56] Haque S., Harries L.W. (2017). Circular RNAs (circRNAs) in health and disease. Genes.

[57] Fan X., Weng X., Zhao Y., Chen W., Gan T., Xu D. (2017). Circular RNAs in cardiovascular disease: An overview. BioMed Res. Int..

[58] Batool S., Javed M.R., Aslam S., Noor F., Javed H.M.F., Seemab R., Rehman A., Aslam M.F., Paray B.A., Gulnaz A.J.P. (2022). Network Pharmacology and Bioinformatics Approach Reveals the Multi-Target Pharmacological Mechanism of Fumaria indica in the Treatment of Liver Cancer. Pharmaceuticals.

[59] Noor F., Rehman A., Ashfaq U.A., Saleem M.H., Okla M.K., Al-Hashimi A., AbdElgawad H., Aslam S.J.P. (2022). Integrating Network Pharmacology and Molecular Docking Approaches to Decipher the Multi-Target Pharmacological Mechanism of Abrus *precatorius* L. Acting on Diabetes. Pharmaceuticals.

[60] Noor F., Tahir ul Qamar M., Ashfaq U.A., Albutti A., Alwashmi A.S., Aljasir M.A. (2022). Network Pharmacology Approach for Medicinal Plants: Review and Assessment. Pharmaceuticals.

[61] Ebbesen K.K., Kjems J., Hansen T.B. (2016). Circular RNAs: Identification, biogenesis and function. Biochim. Biophys. Acta BBA-Gene Regul. Mech..

[62] Hardy J.A., Higgins G.A. (1992). Alzheimer’s disease: The amyloid cascade hypothesis. Science.

